# Unpacking the Emotional Experiences of Learners in a Blended Learning Context

**DOI:** 10.3389/fpsyg.2022.879696

**Published:** 2022-05-25

**Authors:** Shurong Zhao, Junxia Song

**Affiliations:** School of Foreign Languages, Shandong Women’s University, Jinan, China

**Keywords:** blended learning, academic emotion, valence, positive emotion, negative emotion

## Abstract

Understanding the relationship between emotion and learning behavior is conducive to learners’ well-being and effective learning. However, previous studies only regarded emotion as an additional variable, and there lacked specific research on academic emotion in the blended learning (BL) context. BL is characterized by systematic integration of online and face-to-face (F2F) learning, hence leading to special emotional experiences. What is the emotional experience of learners in online learning? What is it like face-to-face? Does the connection between the two have an impact on learners’ emotional experience? In order to address these questions and explore the emotional profiles of learners in BL context, this study constructs a typical BL context in a Chinese university, and conducts questionnaire and focus group interviews with 89 participants at the end of the semester. Data analysis showed that learners’ emotions of face-to-face classes are more intense than those of online learning, both positive and negative. As to positive emotions, paired-sample *t*-test shows that mean values of feeling of challenge, comfort, sense of community, satisfaction, enthusiasm and interest in F2F are significantly higher than those of online learning. About negative emotions, stress, embarrassment, tension and frustration of F2F are significantly stronger than those of online learning, while boredom and disappointment of online learning are more intense than those for F2F section. Theme analysis identified 11 influencing factors of academic emotions, among which degree of difficulty, readiness before class, workload, and interaction are unique to BL and deserve special attention. These findings help form a picture of learners’ academic emotions in BL context. It also provides practical reference for BL course design, so as to inspire emotions which are conducive to effective and in-depth learning.

## Introduction

With ongoing technological transformation and the impact of COVID-19, blended learning (BL) has become the new normal in higher education (Jost et al., 2021). With deepening of researches, the focus of BL exploration has shifted from why to implement BL to how to optimize the design, and further, to how to provide students with meaningful and efficient learning experience (Feng et al., 2018).

Education in the past overemphasized the rational and cognitive functions of the brain and ignored the development of irrational aspects, resulting in emotional illiteracy (Arnold, 1999). With the progress of research in this field, emotion attracts increasing attention as the important role it plays in learning has been recognized (Pekrun et al., 2012). In the process of learning, learners’ emotional state changes, which leads to knowledge acquisition in a spiral format (Kort et al., 2001). Further, emotions stimulate student attention, which in turn stimulates learning behaviors (memory, problem solving, etc.) (Weiss, 2000). Understanding the relationship between emotions and learning behavior is beneficial to students’ mental health, learning outcomes, and interactions (Pekrun et al., 2012; Postareff et al., 2017).

The integration of online and offline learning will inevitably evoke students’ various emotional experiences (Feng X. et al., 2020). Studies have shown that emotions correlate closely with students’ academic performance in BL context (Ramirez-Arellano et al., 2019). Emotions play a critical role in students’ academic interest, engagement, performance, and overall well-being (Riegel and Evans, 2021). However, previous studies in BL context focused on cognition and motivation, attaching little attention to emotion. Limited extant studies on emotion just take it as an additional variable (Henritius et al., 2019), or only focus on emotional engagement [e.g., Gao et al. (2019) and Halverson and Graham (2019)]. There lack studies focusing on emotional experiences of learners in BL context.

Blended learning features the integration of online and face-to-face learning. Learners switch back and forth in the two worlds, online and offline (Ellis, 2014). Therefore, their emotional experiences must be special. As such, what is learners’ online emotional experience like? What is it like in face-to-face section? Does the connection between the two worlds influence their emotional experience? Addressing the above gaps and questions, the present study attempts to explore the emotional profiles of students in BL context. To be specific, it addresses the following research questions:

RQ1. What is learners’ perception of their emotional experiences in BL context?

RQ2. Is there any difference between learners’ emotional experience during online learning and that in face-to-face classes? What are the characteristics of each?

RQ3. What factors influence learners’ emotional experience in BL context?

In addressing these research questions, this study seeks to help form a picture of learners’ academic emotions in the BL context. It also aims to offer practical reference for BL course design, so as to inspire emotions which are conducive to effective and in-depth learning.

## Theoretical Background

### Blended Learning

Blended learning is the thoughtful combination of face-to-face learning and online components, which aims to provide learners engaging learning experience (Garrison and Kanuka, 2004). This kind of integration implies that designing a blended curriculum requires a lot of planning and forethought (Alammary et al., 2014). Halverson and Graham (2019) argued that there is no universal BL framework, while they retain some common features, including increased flexibility and personalization (due to diverse learning approaches), more opportunities for interaction, technical advantages (also accompanied by technical difficulties), increased learning time and abundant learning resources.

Garrison et al. (2001), based on constructivism, proposed the Community of Inquiry (CoI) framework, and believed that there are three key elements that affect BL, namely social presence, pedagogical presence, and cognitive presence. Effective learning occurs when all these three presences are at a high level. Cleveland-Innes and Campbell (2012) added a fourth element to this framework: emotional presence.

As a new learning paradigm, BL does not mean a fixed model. There should be specific and different modes of BL for different grades, different situations, and different goals (Feng et al., 2018). BL also faces multiple challenges. Compared with traditional face-to-face learning, students need to spend more preparation time before class. In addition, it calls for high level of self-regulation and is therefore a major challenge for those with poor self-regulation (Van Laer and Elen, 2017).

People’s understanding of BL has in recent years transferred from the initial discussion of its benefits to the perspective of students. Efforts were taken to create a highly participatory and personalized learning experience for learners (Feng X. Y. et al., 2020). It is believed that the fundamental objective of BL is to enhance student learning experience. In a BL context, students switch back and forth between virtual and real worlds, actively seeking their complementarity (Han and Ellis, 2020). It creates new ways of conceptualizing BL approaches.

Regarding the relationship between BL and emotion, there have been sporadic explorations in the past. Students go through a wide variety of emotional experiences in online and offline learning environments that impact their academic performance and mental health (Feng X. et al., 2020). Halverson and Graham (2019) believe that emotional engagement in BL context can be divided into two dimensions: positive and negative. The former includes enjoyment, happiness and confidence, and the latter covers boredom, frustration, and anxiety. It is usually considered that face-to-face (F2F) section of BL can meet the emotional and social needs of students (Velasquez et al., 2013). But Manwaring et al. (2017) found that F2F helped improve students’ cognitive engagement, while had no significant effect on emotional engagement. These explorations offer hint for further research. However, they did not focus specifically on emotion, or only explored the subdivision perspective of emotional engagement. Exploration on emotion in BL context BL is still scarce.

### Academic Emotions

Emotion is a dynamically evolving mental state, a complex psychological state that involves a subjective experience, a physiological response, and an expressive behavior (Hockenbury and Hockenbury, 2007). It is stimulated by recognizable stimuli. Academic emotion occurs in the learning situation. It refers to various emotional experiences related to students’ activities in the process of teaching and learning, covering a variety of emotions related to learner self-regulation, achievement, personal experience, teaching, and social environment (Pekrun et al., 2002). Measuring students’ academic emotions has profound implications for understanding students’ subjective well-being. Several researches have shown that academic emotion affects students’ mental flexibility, SRL, and academic performance (Pekrun et al., 2002, 2010). Pekrun et al. (2007) identified two dimensions of academic emotion, valence, and activation. Valence refers to whether the emotion is positive or negative. With the activation dimension, emotion falls into two categories, activating and deactivating. These two dimensions divide emotions into four kinds, namely positive activating, positive deactivating, negative activating, and negative deactivating. Generally speaking, positive emotions are more beneficial to learning than negative emotions (Postareff et al., 2017). However, this is not always the case. The relationship between emotion and learning is far more complex than what it is supposed to be. For instance, stress and anxiety seem negative, but both are beneficial to learning in some cases (Pekrun, 2006). Academic emotion can also be categorized as trait and state, which are often intertwined with each other (Cheng et al., 2020). This study focuses on the valence of emotion in the context of BL. Most studies explore emotion as a feature rather than as a state (Henritius et al., 2019), while this study focuses on the emotional state in BL context.

Academic emotion is closely related to the teaching and learning process. Attention, self-regulation, and motivation are all mediating factors that affect students’ emotion and achievement pursuit in learning (Henritius et al., 2019). Pekrun (2011) held that emotions are triggered by proximal antecedents and distal antecedents. Proximal antecedents include control appraisals and value appraisals. The former refers to learners’ evaluation of whether he or she can control the learning activities, and the latter refers to learners’ evaluation of the importance of learning outcome. Distal antecedents include personality, social factors, and cultural factors.

Blended learning involves two completely different situations, online and offline. In this special situation, students go through a variety of emotional experiences. Teachers and course designers should understand learners’ academic emotions and take effective interventions to improve their mental well-being and enhance pleasant learning experience.

## Research Methodology

To explore the emotional experience of learners in BL context, this study adopted mixed methods, trying to obtain rich and comprehensive data (Gass and Mackey, 2015). Questionnaires on emotion were conducted to get quantitative data. Focus group interviews were carried out to acquire qualitative data with the aim of identifying influencing factors, discovering personal feelings of the participants and the stories behind the questionnaire. Quantitative and qualitative data corroborate each other to ensure the validity of research findings and their interpretation.

### Context of the Study

This research was conducted in Business English course at a Chinese university. We constructed a typical BL environment which integrates online and F2F learning. In the online learning stage, students watch videos online and work in pairs or groups of three to complete matching tasks. In the F2F learning section, a variety of learning activities are organized to practice and consolidate the content learned and facilitate deep learning. Online and F2F learning are carried out in sequence, that is, one section of online learning is followed by a F2F class, then followed by another online. Implementation of BL lasted for a whole semester (16 weeks), the detailed information of which is shown in Table 1. The two instructors of this course have BL implementation experience of more than 5 rounds, with clear grasp of BL’s connotation and rich experiences in BL course design and implementation. All the learners of the course are also the participants in this study. They were 89 business English majors, from two classes, aged 19–23, all with prior BL experience.

**TABLE 1:** Basic information of the blended learning (BL) context.

Class frequency and time	Twice a week, 90 min per session
Total credits	4
Number of chapters	8
Number of instructors	2
Number of students	89 (in pairs or in groups of three)
Online resources	Recorded videos covering all chapters
Online learning	Watching online videos and completing matching tasks in pairs or in groups of three
Face-to-face learning	Practice, presentation, explanation, and collaborative work

### Questionnaire

The questionnaire of this study was adapted from the emotion scale of Hakkarainen et al. (2007). It consists of four parts. The first part is about demographic information. The second part covers 20 5-point Likert scale items of 20 emotions (nine positive and 11 negative) for the F2F section, asking the participants to indicate a degree of emotional state (1 = not at all, 5 = to a great extent). The 20 emotions include comfort, sense of community, feelings of challenge, relief, joy, trust, satisfaction, enthusiasm, interest, stress, embarrassment, dispiritedness, boredom, disappointment, feelings of inadequacy, tension, irritation, worry, frustration, and uncertainty. The third part is about the same 20 emotional scale items, but about online learning. The fourth part includes three Likert scale items (1 = strongly disagree, 5 = strongly agree) on the overall emotional experience of participants. The integration of online and F2F paradigm lasts for the whole semester, thus providing a stable BL context. The questionnaire was administered at the end of the semester (the sixteenth week), inquiring the participants about their feelings when looking back from week 1 to week 16. 88 effective questionnaires were recovered.

It is worth noting that after each F2F class and each time of online learning in this semester, students were asked to mark the above-mentioned 20 emotion scales in paper-form questionnaire (the data will be analyzed in another paper). Therefore, learners are deeply impressed by their emotional state of face-to-face classes and online learning. Before filling the questionnaire for the first time, instructors offered sample illustrations of the 20 emotions, discussed with students the meaning of these emotions in detail and reached consensus, so that students’ understanding of emotions was clear and homogeneous. After many times of marking the emotional scale, learners are fully prepared for filling out the emotional scale questionnaire at the end of the semester. This approach eliminates inaccurate data due to misunderstandings and accidental factors, and ensures the validity.

Data of the questionnaire were analyzed with SPSS 22.0. The Cronbach’s α of the questionnaire was 0.909, and that of each dimension was above 0.875, indicating high degree of internal consistency and reliability. Descriptive analysis was used to explore the general state of emotional experiences, online and face-to-face. Paired-sample *t*-test was used to find differences between emotional state of online learning and that of face-to-face section.

### Focus Group Interview

Focus group interview was adopted to obtain deep insights into the emotional profile of learners in the BL context and to identify the factors that affect their emotional states. The advantage of focus group interview lies in its potential of providing rich qualitative data (Glesne, 2016).

According to the academic performance of the previous semester, all 89 students were divided into high-score, medium-score and low-score groups. Two students were randomly selected from each of these three groups to form a focus group. Finally, six focus groups were selected (36 students). The interview questions are as follows:

FG1. What was your emotional state like during face-to-face learning? What was it like during online learning?

FG2. Looking back on the emotional experience of learning throughout the semester, have there been ups and downs? Is there any emotional experience that particularly impressed you? Please cite an example.

FG3. What factors influence your emotional experience during the online and F2F learning?

The interviews were recorded after getting consent of the respondents. After the interview outline was drawn up, it was tested and revised accordingly. The interviews were conducted by two researchers, with minimum guidance for respondents. Only additional questions were asked to lead the topic further, or additional questions were asked to clarify what the respondents wanted to express. To avoid language barriers, interviews were conducted in Chinese, the native language of the respondents.

The focus group interviews lasted 16–25 min. After that, one researcher performed the transcription and the other checked it. Thematic analysis was then conducted, which went through three stages: initial coding, focused coding, and axial coding. In the initial coding stage, the participants’ recordings were analysed sentence by sentence and the themes were identified; in the focused coding stage, some original themes were corrected and some new themes were added; in the axial coding stage, the connections among the themes were found. Two researchers analysed the transcription independently, and differences were discussed until all disputes were agreed upon.

## Findings

### Students’ Perception of Their Emotional Experience in Blended Learning Context

Regarding learners’ overall emotional experience in BL context (in answering research question 1), paired-sample *t*-test of the questionnaire data showed that the mean values of learners’ positive emotions were significantly higher than those of their negative emotions, both for the face-to-face section (*t* = 11.854, *p* < 0.01) and online learning (*t* = 11.455, *p* < 0.01) (Table 2). It suggests that students’ overall emotional experience in a BL setting is positive.

**TABLE 2:** Comparison of positive emotions and negative emotions (paired *t*-test).

Items	Paired (M ± SD)		Mean difference (Paired 1-Paired 2)	*t*	*p*
	Paired 1	Paired 2			
F2F-positive *Paired* F2F-negative	4.00 ± 0.60	2.69 ± 0.77	1.31	11.85	0.000**
OL-positive *Paired* OL-negative	3.81 ± 0.61	2.57 ± 0.80	1.24	11.45	0.000**

**p < 0.01. N = 88. OL, online; F2F, face-to-face.

**FIGURE 1:**
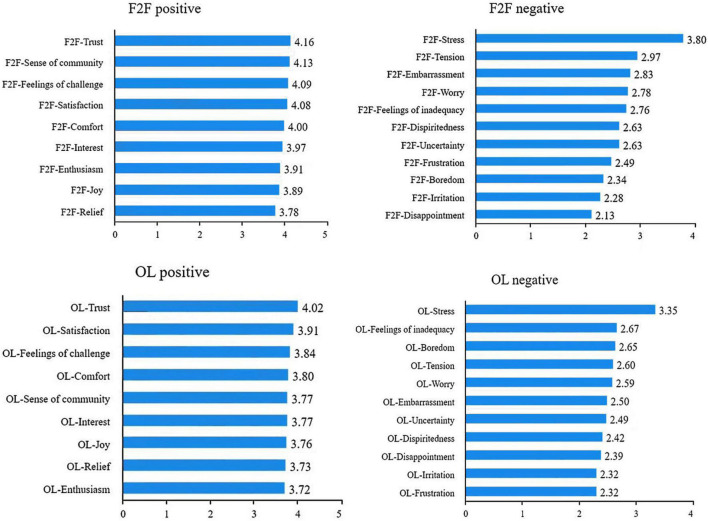
Detailed emotional experience for F2F and online learning (each sorted by mean value). OL, online; F2F, face-to-face. *N* = 88.

As to the detailed emotional experience of learners in BL context (Figure 1), descriptive analysis shows that for the face-to-face section, the top three learners’ positive emotions in terms of mean values are trust, sense of community and feelings of challenge. The top three negative emotions are stress, tension and embarrassment. For online learning, the three positive emotions are trust, satisfaction and feeling of challenge, and the top three negative emotions are stress, feelings of inadequacy, and boredom.

Regarding the fluctuation of the learners’ emotional state, frequency analysis shows that 65.9% of the students agree or strongly agree the statement of “My emotional state of learning went through ups and downs.” In the focus group interview, student S6 said, “at the beginning of the semester, I was enthusiastic and excited for learning, and then was slack in the middle term. Toward the end of the term, I felt sense of urgency and inadequacy.” Student S12 mentioned, “I was very nervous during the question-and-answer session in classroom learning, but relaxed in online learning.” S25 said, “I experienced ups and downs in my emotional state. When I was learning about L/C, I felt that I didn’t understand very well and was very anxious. After continuous study and with the help of my classmates, I feel that everything is getting better.”

### Comparison of Emotional Experience Between Face-to-Face Section and Online Learning

In order to address research question 2, paired sample *t*-test was used to compare the positive emotion of F2F and that of online learning, and to compare the negative emotion of these two sections. Data analysis (Table 3) shows that the mean values of positive emotions of F2F are significantly higher than those of online learning (*t* = 3.393, *p* < 0.05), so do those of negative emotions (*t* = 2.387, *p* < 0.05). As such, a tentative conclusion can be drawn that the emotions of face-to-face classes are more intense than those of online learning, both positive and negative.

**TABLE 3:** Comparison between face-to-face (F2F) and online emotions.

Items	Paired (M ± SD)		Mean difference (Paired 1-Paired 2)	*t*	*p*
	Paired 1	Paired 2			
F2F-positive *Paired* OL-positive	4.00 ± 0.60	3.81 ± 0.61	0.19	3.393	0.001**
F2F-negative *Paired* OL-negative	2.69 ± 0.77	2.57 ± 0.80	0.12	2.387	0.019*

*p < 0.05, **p < 0.01.

As to the detailed emotions comparison, we first compared the 9 positive emotions regarding F2F with those of online learning. Paired-sample *t*-test shows that the mean values of feeling of challenge, comfort, sense of community, satisfaction, enthusiasm, and interest in F2F are significantly higher than those of online learning (Table 4). There is no significant difference in the remaining three positive emotions.

**TABLE 4:** Comparison of positive emotions between face-to-face (F2F) and online sections.

Items	Paired (M ± SD)		Mean difference (Paired 1-Paired 2)	*t*	*p*
	Paired 1	Paired 2			
F2F-Feelings of challenge *Paired* OL-Feelings of challenge	4.09 ± 0.80	3.84 ± 0.90	0.25	2.534	0.013*
F2F-Comfort *Paired* OL-Comfort	4.00 ± 0.82	3.80 ± 0.83	0.20	2.751	0.007**
F2F-Sense of community *Paired* OL-Sense of community	4.13 ± 0.87	3.77 ± 0.99	0.35	3.248	0.002**
F2F-Relief *Paired* OL-Relief	3.78 ± 0.93	3.73 ± 0.94	0.06	0.560	0.577
F2F-Joy *Paired* OL-Joy	3.89 ± 0.89	3.76 ± 0.88	0.13	1.182	0.240
F2F-Trust *Paired* OL-Trust	4.16 ± 0.66	4.02 ± 0.68	0.14	1.979	0.051
F2F-Satisfaction *Paired* OL-Satisfaction	4.08 ± 0.73	3.91 ± 0.72	0.17	2.237	0.028*
F2F-Enthusiasm *Paired* OL-Enthusiasm	3.91 ± 0.84	3.72 ± 0.84	0.19	2.024	0.046*
F2F-Interest *Paired* OL-Interest	3.97 ± 0.78	3.77 ± 0.88	0.19	2.187	0.031*

*p < 0.05, **p < 0.01. N = 88.

The 11 negative emotions regarding F2F and online learning were also compared (Table 5). Paired-sample *t*-test shows that the mean values of stress, embarrassment, tension and frustration of F2F are significantly higher than those of online learning (*p* < 0.05), indicating that these emotions are stronger during F2F learning. Data of focus group interview echoed this result. Thirteen students referred to their emotional state of stress during question-and-answer and quiz time and nine students mentioned their tension in class. On the other hand, the mean values of boredom and disappointment of online learning are significantly higher those for F2F section (*p* < 0.05), indicating that these emotions are more intense during online learning.

**TABLE 5:** Comparison of negative emotions between face-to-face (F2F) and online sections.

Items	Paired (M ± SD)		Mean difference (Paired 1-Paired 2)	*t*	*p*
	Paired 1	Paired 2			
F2F-Stress *Paired* OL-Stress	3.80 ± 0.98	3.35 ± 1.06	0.44	3.544	0.001**
F2F-Embarrassment *Paired* OL-Embarrassment	2.83 ± 1.09	2.50 ± 1.04	0.33	2.921	0.004**
F2F-Dispiritedness *Paired* OL-Dispiritedness	2.63 ± 1.15	2.42 ± 1.01	0.20	1.949	0.055
F2F-Boredom *Paired* OL-Boredom	2.34 ± 1.04	2.65 ± 1.02	–0.31	–2.988	0.004**
F2F-Disappointment *Paired* OL-Disappointment	2.13 ± 1.00	2.39 ± 0.96	–0.26	–3.029	0.003**
F2F- Inadequacy *Paired* OL-Inadequacy	2.76 ± 1.04	2.67 ± 1.06	0.09	0.956	0.342
F2F-Tension *Paired* OL-Tension	2.97 ± 1.07	2.60 ± 1.02	0.36	3.145	0.002**
F2F-Irritation *Paired* OL-Irritation	2.28 ± 1.08	2.32 ± 0.94	–0.03	–0.410	0.683
F2F-Worry *Paired* OL-Worry	2.78 ± 1.15	2.59 ± 1.09	0.19	1.805	0.075
F2F-Frustration *Paired* OL-Frustration	2.49 ± 0.99	2.32 ± 0.92	0.17	2.143	0.035*
F2F-Uncertainty *Paired* OL-Uncertainty	2.63 ± 1.03	2.49 ± 1.02	0.14	1.618	0.109

**p < 0.01. N = 88.

### Factors Influencing Learner’ Academic Emotion

As to the influencing factors of academic emotion (research question 3), focus group interview offered detailed and rich data. Thematic analysis identified 11 influencing factors, including degree of difficulty, readiness before class, mastery of knowledge, workload, learning content, teaching paradigm, personal emotion, interaction and collaboration, assessment, peer influence, and self-regulation (Table 6). Degree of difficulty means that difficult learning content leads to negative emotional experiences (e.g., anxiety, frustration, and nervousness), while easy content promotes distraction. For readiness before class, learners hold that if they are fully prepared before the class (online learning and review tasks have been completed), they will be full of enthusiasm and acquire a lot during the class. Mastery of knowledge means that if they have acquired adequate background knowledge and know well about the topic of that class, they experience emotional state of confidence, enthusiasm, and satisfaction. For workload, too many online and offline learning tasks make learners feel dispirited, or even annoyed. Learning content also influences their emotional state. If it is interesting and challenging, they feel excited and ready to learn. But some long videos and uninterested topics bring about feelings of boredom. The teaching paradigm brings emotional ups and downs. The face-to-face class brings tense and stressful feelings. However, learners have strong sense of gain and sense of community. Online learning is easy and flexible, but sometimes learners can’t pluck up spirit. Personal mood in learner’ daily life influences their emotional state during classroom and online learning. Regarding interaction and collaboration, smooth and harmonious teacher-student and student-student interaction and collaboration result in positive emotions. Regarding assessment, many learners feel nervous and stressed during the question time and quiz in class, but they also think that it urges them to truly acquire knowledge. Peer influence means that excellent performance of peers brings pressure, and peer help leads to relief. Self-regulation brings peace of mind and pleasure as they are well-prepared before class.

**TABLE 6:** Theme analysis of influencing factors for emotional state.

Themes	Frequency counts	Representative quotation
Degree of difficulty	14	Learning content in online leaning is very difficult, with many unfamiliar specialized terms. As a result, I tend to get distracted. (S15) The more difficult the content, the more miserable my emotional experiences turned to be. (S17)
Readiness before class	11	If the content of this class is easy to understand and I have fully prepared before the class, I will be more emotionally motivated to learn in the face-to-face class. But with difficult content and insufficient preview, I feel low spirit, irritation and distraction in the face-to-face class. (S3)
Mastery of knowledge	11	The experience of learning Letter of credit impressed me deeply. Although, the content is difficult, I am familiar with the relevant background knowledge. I felt excited when I suddenly came across something I had learned before. (S30)
Workload	10	When there is a lot of homework, many tasks, and difficult video content to learn, I have to replay the videos time and time again. It makes me annoyed. (S25)
Learning content	9	If the learning materials and videos given in class are interesting, I will be interested in learning this chapter. (S17)
Teaching paradigm	9	In the face-to-face class, I concentrate on teacher’s explanation and learning activities, but I am very nervous. Online learning is much easier and more casual. The difference between the two sections is significant. (S19)
Personal emotion	9	When being in a good mood, I am more motivated to learn and willing to participate in class interaction. Conversely, when I am troubled by some bad mood, my concentration decreases. (S10)
Interaction and collaboration	8	When there is little interaction in the classroom, I feel dispirited and lack of sense of community. (S11) I feel pair-work valuable. Meeting difficulty in learning, we two can discuss it first, and then listen attentively to teacher’s explanation on it during the face-to-face class. (S15)
Assessment	8	I have love-hate feelings toward question time and quiz. It is stressful but it does have an urging effect. (S32) I was joyful and excited when getting A + for my homework. (S2)
Peer influence	5	When other students have mastered the learning content but I do not yet, I feel very anxious and frustrated. (S23)
Self-regulation	4	Compared with face-to-face classes, online learning is easier and more flexible. My online learning performance depends to a large extent on self-regulation, but it is inevitable that sometimes there will be lazy thoughts. (S1)

*S, student N = 36.*

## Discussion and Implications

### On Emotional Experiences of Learners in Blended Learning Context

It is found in this study that the mean values of learners’ positive emotions were significantly higher than those of negative emotions, and hence the students’ overall emotional experience in the BL context was positive. One of the advantages of BL is that students can learn online at their own pace, providing a safe and relaxing environment. This safe setting allows students to learn to deal with difficult emotions. The integration of online and F2F brings about flexible learning space, in which personalized needs are met, hence leads to reassurance and confidence.

This study found that in the F2F section, the top three learners’ positive emotions are trust, sense of community, and feelings of challenge. The top three negative emotions are stress, tension, and embarrassment. In Online learning, the top three positive emotions are trust, satisfaction and feeling of challenge, and the top three negative emotions are stress, feelings of inadequacy, and boredom. These findings are slightly different from previous studies. Hara and Kling (1999) report that frustration, isolation, anxiety, and confusion are the most commonly experienced emotions by students in online learning. The difference may lie in the inherent characteristics of the BL context. As such, the emotional experience in BL is surely different from that of pure online learning. Further, this study sorts positive emotions and negative emotions of online and F2F sections respectively, which facilitates in-depth and comprehensive understanding of students’ emotional profile, hence targeted improvement of teaching design can be achieved.

Regarding positive and negative emotions, on the one hand, previous studies have reported a positive relationship between positive emotions and academic achievement (Postareff et al., 2017). On the other hand, Cheng et al. (2020) argue that negative emotion may be more conducive to long-term learning, and that it leads to real learning. Negative emotions lead to concentration (McConnell and Eva, 2012), while positive emotions promote distraction (Dreisbach and Goschke, 2004). Findings of this study, for instance, learners’ complex love-hate emotions about question time and test which create tension and stress for them, confirmed the complexity of the relationship between emotion and learning.

### On Comparison of Emotions Between Face-to-Face and Online Learning

Data analysis showed that the emotions of face-to-face classes are more intense than those of online learning, both positive and negative. This finding echoed Marchand and Gutierrez (2012) in that online and face-to-face academic emotions are significantly different. Regarding the comparison of specific emotions, data analysis indicated that the positive aspect of F2F lies in sense of challenge and community, but learners are more stressed. Online learning features relaxing atmosphere, but learners’ feelings of boredom and disappointment are stronger than those in F2F section. This contrast may also stem from the respective characteristics of F2F and online learning. To make the best of the two worlds (Osguthorpe and Graham, 2003) and improve learners’ emotional experience, differentiated emotional support based on BL characteristics should be implemented which targets individual learner’s needs. Regarding support, failure to provide learning strategy guidance in challenging units will make students feel frustrated (Strayer, 2012). The teacher-student space separation in online learning usually results in lack of emotional support from teachers (Dang et al., 2019), and leads to learning burnout. Teachers should create learning environments where emotional safety is valued and respected, which is beneficial for students who are concerned about their performance. Further, measures should be adopted to create a positive learner identity so that learner motivation can be maintained. Efforts can also be made to provide a supportive learning community in which learners have opportunities of self-expression and self-perception. To sum up the above, teachers should provide differentiated emotional support that fits the characteristics of BL in a timely manner, thereby ensuring task participation, harmonious relationships, self-confidence, security, and achievement.

### On Factors Influencing Learners’ Academic Emotions

Theme analysis of focus group interview identified 11 factors affecting academic emotion in BL context, including degree of difficulty, readiness before class, mastery of knowledge, workload, learning content, teaching paradigm, personal emotion, interaction and collaboration, assessment, peer influence, and self-regulation. Among them, factors including degree of difficulty, readiness before class, workload, and interaction are unique to BL and deserve special attention.

The most frequently mentioned factor is degree of difficulty. The reason may be that cognitive imbalances in students may create opportunities for deep learning of difficult content; however, if students fail to regain their balance, this state may result in frustration and boredom (D’Mello and Graesser, 2012). It is also related to the degree of control students have over the learning content. Riegel and Evans (2021) found that students’ positive emotion in quiz was stronger than that in test, possibly because learners thought quiz was more controllable. Students enjoy it when they are interested in a topic and feel in control of the task, and they feel frustrated when they lack control over it (Ramirez-Arellano et al., 2019). In addition, high-quality curriculum resource constitutes a key to the success of BL. Therefore, we suggest that course designers provide high-quality resources that are in line with students’ cognitive level, so as to stimulate positive emotions and suppress high-level negative emotions. To be specific, when selecting online resources, factors like user-friendliness, perceived community, richness of content, difficulty and perceived flexibility should be taken into account (Lu and Chiou, 2010).

Readiness before class is also an important factor unique to BL. The effectiveness of BL depends heavily on the students’ engagement in pre-class activities (Zhao and Li, 2021), and in-class learning performance is largely determined by pre-class preparation (Rahman et al., 2015). From pedagogical perspective, teaching, and learning in the BL context is highly unstable and fluctuating, so it is critical to maintain seamless connection between F2F and online learning. Hence targeted measures and learner strategy development should be conducted, which may include matching evaluation and supervision, group collaboration and SRL training.

Interaction, collaboration, and workload also deserve attention. Interaction and evaluation in the learning community can affect students’ emotions (Bock et al., 2005). According to “interactions as transactions” put forward by Wagner (2006), interaction among participants is an important guarantee for an effective BL. In in online learning section of BL, students have less interaction with classmates and teachers, and feel less engaged and isolated (Dang et al., 2019). On the other hand, tensions that exist in group activities can provide more opportunities for interaction and generate new practices. Therefore, developing well-designed collaborative activities that effectively stimulate multi-dimensional interactions becomes a key to effective BL. As for workload, this study shows that it is an important source of negative emotions. Heavy workload has always been one of the troublesome problems for BL. As such, controlling the workload within an acceptable level is also a must for BL designers when arranging various learning activities.

## Limitations

It is acknowledged that this study has certain research limitations. First, the data on emotional experience in this study comes from the self-reports and subjective feelings of learners. Future research may combine self-reports with learning analytics with modern technologies, such as facial expression recognition. Second, participants in this study are all from one discipline, which is insufficient for the generalization of results. Similar inquiries can be conducted in multiple disciplines in the future to form a more comprehensive picture of the emotional profile of learners in BL context.

## Conclusion

Blended learning features the systematic integration of online and face-to-face learning. Learners switch back and forth in the two worlds, online and offline. Therefore, their emotional experiences are special. This study sought to explore learners’ emotional experience in a BL context. By conducting questionnaire and focus group interview, the following findings were discovered. First, students’ overall emotional experience in the BL context was positive and they experienced ups and downs in their emotional state. Second, the emotions of face-to-face classes are more intense than those of online learning, both positive and negative. The positive aspect of F2F lies in sense of challenge and community, but learners are more stressed. Online learning features relaxing atmosphere, but learners’ feelings of boredom and disappointment are stronger than those in F2F section. Third, 11 influencing factors of academic emotions were discovered, among which degree of difficulty, readiness before class, workload, and interaction are unique to BL and deserve special attention. These findings offer detailed evidence for what learners’ academic emotions are like in the BL context. It also provides practical reference for course designers in designing online or face-to-face learning activities, in development of learning resources and in cultivating learning strategies, so as to inspire emotions which are conducive to effective and in-depth learning.

With the acceleration of technological transformation, BL takes on more diverse forms, and its connotation continues to expand. The exploration of learners’ emotional state under BL situation also needs to be deepened and expanded. Future research can be expanded to more disciplines, and new learning analytic technologies such as facial expression recognition and eye movement can be adopted to form a more objective and comprehensive picture of learners’ emotional profiles.

## Data Availability Statement

The raw data supporting the conclusions of this article will be made available by the authors, without undue reservation.

## Ethics Statement

The studies involving human participants were reviewed and approved by Shandong Women’s University. The patients/participants provided their written informed consent to participate in this study.

## Author Contributions

SZ drafted the manuscript. JS revised and proofread the manuscript. Both authors conducted the theme analysis of focus group interview, contributed to the study design, and approved the final version of the manuscript for submission.

## Conflict of Interest

The authors declare that the research was conducted in the absence of any commercial or financial relationships that could be construed as a potential conflict of interest.

## Publisher’s Note

All claims expressed in this article are solely those of the authors and do not necessarily represent those of their affiliated organizations, or those of the publisher, the editors and the reviewers. Any product that may be evaluated in this article, or claim that may be made by its manufacturer, is not guaranteed or endorsed by the publisher.
