# Supplementary material for: Unpacking the Emotional Experiences of Learners in a Blended Learning Context
Source: Front Psychol. 2022 May 25;13:879696. doi: 10.3389/fpsyg.2022.879696 (PMC9174991; doi:10.3389/fpsyg.2022.879696)
Supplement: Supplementary file 1 [file Data_Sheet_1.docx]

Appendix 1

Questionnaire for learners’ emotional experiences in BL context

1. My emotional experiences during ***face-to-face learning***.

| Dimension | Emotions | Scale (1 = not at all, 5 = to a great extent) | | | | |
| --- | --- | --- | --- | --- | --- | --- |
|  |  | 1 | 2 | 3 | 4 | 5 |
| Positive | Feelings of challenge |  |  |  |  |  |
|  | Comfort |  |  |  |  |  |
|  | Sense of community |  |  |  |  |  |
|  | Relief |  |  |  |  |  |
|  | Joy |  |  |  |  |  |
|  | Trust |  |  |  |  |  |
|  | Satisfaction |  |  |  |  |  |
|  | Enthusiasm |  |  |  |  |  |
|  | Interest |  |  |  |  |  |
| Negative | Stress |  |  |  |  |  |
|  | Embarrassment |  |  |  |  |  |
|  | Dispiritedness |  |  |  |  |  |
|  | Boredom |  |  |  |  |  |
|  | Disappointment |  |  |  |  |  |
|  | Feelings of inadequacy |  |  |  |  |  |
|  | Tension |  |  |  |  |  |
|  | Irritation |  |  |  |  |  |
|  | Worry |  |  |  |  |  |
|  | Frustration |  |  |  |  |  |
|  | Uncertainty |  |  |  |  |  |

1. My emotional experiences during ***online learning.***

| Dimension | Emotions | Scale (1 = not at all, 5 = to a great extent) | | | | |
| --- | --- | --- | --- | --- | --- | --- |
|  |  | 1 | 2 | 3 | 4 | 5 |
| Positive | Feelings of challenge |  |  |  |  |  |
|  | Comfort |  |  |  |  |  |
|  | Sense of community |  |  |  |  |  |
|  | Relief |  |  |  |  |  |
|  | Joy |  |  |  |  |  |
|  | Trust |  |  |  |  |  |
|  | Satisfaction |  |  |  |  |  |
|  | Enthusiasm |  |  |  |  |  |
|  | Interest |  |  |  |  |  |
| Negative | Stress |  |  |  |  |  |
|  | Embarrassment |  |  |  |  |  |
|  | Dispiritedness |  |  |  |  |  |
|  | Boredom |  |  |  |  |  |
|  | Disappointment |  |  |  |  |  |
|  | Feelings of inadequacy |  |  |  |  |  |
|  | Tension |  |  |  |  |  |
|  | Irritation |  |  |  |  |  |
|  | Worry |  |  |  |  |  |
|  | Frustration |  |  |  |  |  |
|  | Uncertainty |  |  |  |  |  |

1. Overall emotional experience during this whole term (1 = strongly disagree, 5 = strongly agree).

| Items | 1 | 2 | 3 | 4 | 5 |
| --- | --- | --- | --- | --- | --- |
| My emotional experience was relatively stable throughout the semester. |  |  |  |  |  |
| I have had ups and downs in my emotional experience throughout the semester in this course. |  |  |  |  |  |
| My overall emotional experience throughout the semester in this course was pleasant and positive. |  |  |  |  |  |

Appendix 2

Sample illustrations of the 20 emotions

| Dimension | Emotions | Sample illustration |
| --- | --- | --- |
| Positive | Feelings of challenge | For me, the face-to face/online learning calls for great efforts and this process leads to progress. |
|  | Comfort | I feel comfortable when learning online/face-to-face. |
|  | Sense of community | I considered myself being in a community with my classmates and teacher when learning online/face-to-face. |
|  | Relief | When learning online/face to face, I feel relief or freed. |
|  | Joy | I enjoy attending the class/learning online. |
|  | Trust | I feel being trusted when participating the face-to-face/ online learning. |
|  | Satisfaction | I’m satisfied with the face-to-face/online learning. |
|  | Enthusiasm | I’m enthusiastic when participating the online/face-to-face learning activities. |
|  | Interest | I’m interested in the content of class and activities. |
| Negative | Stress | I get great worry caused by difficult situation. |
|  | Embarrassment | I feel embarrassed when being unable to answer teacher’s questions or giving unsatisfactory answers. |
|  | Dispiritedness | I feel myself in low spirit when taking the class or learning online. |
|  | Boredom | Studying for my course bores me. |
|  | Disappointment | I feel disappointed during or after the face-to-face/online learning. |
|  | Feelings of inadequacy | I often feel inadequacy of my business knowledge or language proficiency. |
|  | Tension | I feel very nervous when learning face-to-face/online. |
|  | Irritation | Studying makes me irritated. |
|  | Worry | I often worry about my performance or a difficult situation in face-to-face /online learning. |
|  | Frustration | I feel frustrated during or after the class/online learning. |
|  | Uncertainty | I feel uncertain about my performance or the learning effect. |
